# The Treatment of Cough as a Symptom in Phthisis

**Published:** 1893-07-22

**Authors:** 


### The Hospital Clinic.

[*The Editor will be glad to receive offers of co-operation and contributions from members of the profession. All letters should be addressed to* The Editor, The Lodge, Porchester Square, London, W.]

### ROYAL HOSPITAL FOR DISEASES OF THE CHEST, CITY ROAD, E.C.*

### The Treatment of Cough as a Symptom in Phthisis.

In dealing with this most troublesome symptom it is necessary at the outset to bear in mind that to a large extent coughing is nature's method of getting rid of material which if left in the air passages, impedes the free passage of air to or from the lungs, and, further, not only acts as an irritant, but also is apt to decompose and so add to the morbid processes already going on in the pulmonary tissues. It becomes therefore important to recognise when art may interfere with a natural process, and when this may be left alone. Certain limits may be readily defined—thus, when coughing leads to vomiting, and so interferes with digestion, clearly some interference is necessary; or again, when sleep is much disturbed. Further, it is right to interfere when it is possible by lessening the amount of material to be expectorated to diminish the frequency of the cough, or when cough depends on irritability of some part of the air passages, and is excessive in frequency or in each paroxysm, when compared with the actual amount of material brought up.

There is therefore a fairly wide field within the limits of which it is justifiable and necessary to interfere with cough, and there is a considerable variety of remedies or therapeutic agents which are found useful and satisfactory for this purpose.

Taking first the very common cough, which comes on either immediately on getting into bed or within a short time, this is often met by the use of pil ipecac. opiat c scilla (pulv. ipecac. gr. ss, pulv. ipecac. co. gr. ij., pil scill. co. gr. i., ext. aloes gr. 1-6) given shortly before bed time. If the cough continue during the night, and the phlegm seems tough and difficult to raise, lozenges containing extract of liquorice and oil of anise are often very useful in lessening cough and promoting easy expectoration. These particular lozenges are very extensively used in the hospital, and in many cases afford great relief, especially when given for a dry hacking cough, which is causing the patient to become worried and exhausted, yet is not effectual in clearing the air passages. Other lozenges used frequently are (1) those containing pulv. acaciæ with tragacanth, which are mainly demulcent, and so useful in cases where there is irritation about the fauces and upper part of the throat, and (2) those containing extract of liquorice and compound powder of tragacanth, with 1-36 of a grain of hydrochlorate of morphia in each. This latter form of lozenge will often ease a troublesome cough coming on during the night, and so will enable the patient to get to sleep. When the cough is mainly due to irritation of the bronchi, and is accompanied by profuse secretion, not especially nummular in character, inhalations are found useful—either tinct. benz. co. from a steam inhaler (ʒi of tincture to oj of water), or creasote or eucalyptus on a Burney Yeo's respirator. The latter inhalation contains, in addition to oil of eucalyptus (ʒii.), both tr. benz. co. (ʒiii.), and thymol (ʒi.), with sp. chloroformi (ad. ʒi.), and is also useful in diminishing the secretion from and disinfecting cavities in the lungs, by this means checking the cough also.

Morphia is used sparingly, and in small doses, and at once stopped if it appears to interfere with digestion. Two forms of linctus containing this drug are in use, viz., linct. morph., which is made up of morph. hydrochlor. grain 1-16, ac. hydrochlor. dil. ♏i., syr. scill, ♏xx., ac. hydrocyan, dil. ♏ij., aq. ad. ʒi., and (2) linct. morph. rub.r (so-called from its colour), which contains ac. tartar ʒi., vin. ipecac, ʒi., liq. morph. acetat ʒiss., and syr. rhœados ad ℥ii.

The latter is the more agreeable to take, and though checking irritation, yet assists moderate expectoration, and is thus useful when the patient has great difficulty in getting rid of thick, sticky mucus.

Both forms of linctus are given in drachm doses not more than once or twice during the day, but during the night up to three or four times if necessary, allowing two or more hours to elapse between the doses.

When cough is accompanied by much pain, which is commonly referred either to the region of the apices of the lungs or also to the sternum or the region of the bases, counter irritation frequently affords much relief. It is mostly effected either by iodine painted on, or small blisters (2×2) or by hot fomentations. Pain referred to the bases is generally relieved by strapping the side, as described in a previous paper.

One of the most intractable forms of cough is that in which the paroxysms are prolonged and end in retching or vomiting. As a rule neither linctus nor lozenges will relieve these, and other remedies must be tried. Perhaps the best results are obtained from the inhalation of one, two, or three drops of chloroform from a Burney Yeo's respirator; this at times acts like a charm when given at the commencement of a fit of coughing, but of course the relief is only temporary. Spraying the fauces and pharynx with cocaine affords at times a great relief, or conium may be inhaled from a steam inhaler (succ. conii ʒij., liq. ammon. ♏x). This also is valuable in relieving pain and irritation in the throat, which tend to keep up the cough. Sometimes the simple sucking of a little ice or the sipping of hot-water in teaspoonfuls will prevent retching or vomiting.

For the continual cough and profuse expectoration accompanying extensive cavitation, the remedies commonly prescribed are the inhalations of eucalyptus or creasote already mentioned, or a third form containing three grains of iodine with two drachms each of æther and carbolic acid, made up with rectified spirit to the ounce, thus resembling Coghill's formula. Of any of these, 10 drops are put on the wool in a respirator, which is worn as continuously as maybe, either for alternate half hours, if, as occasionally happens, some headache is caused, or more continuously still.

As the general health improves under constitutional treatment by cod liver oil, tonics, and similar remedies, it is found, as might be expected, that cough diminishes, so that what has been said in a previous paper on the treatment of phthisis in general applies also to the treatment of cough in particular. Especially is this true of cod liver oil, which, as in chronic bronchitis, seems to exert in some cases almost a specific influence over cough.

There still remains one considerable cause of cough to be touched upon, viz., laryngeal phthisis, and under this head our remarks must necessarily apply rather to general treatment of this variety of tubercular affection than to the special treatment of the cough it causes.

In the first place, everything that has been said on the general treatment of phthisis in a former article applies with equal force to the treatment of this complication, but, in addition, certain local remedies have been found of great value. Chief among these is lactic acid. A solution of this drug is applied by means of a brush to the larynx once a day regularly for some

* By the Resident Medical Officer.

weeks. We generally begin with a solution of 10 or 15 per cent., going on after a week or two to a solution of 20 per cent. Under this treatment ulceration is arrested, and a certain amount of contraction of the tissues sets in, pain is lessened, and with the disappearance of irritation the cough is quieted. This is especially marked in early cases, but even those of long standing yield to treatment, and the patient experiences much relief.

In cases where there is great pain, with extensive and deep ulceration, an insufflation daily of a powder containing morph. hydrochlor., gr. $\frac{1}{8}$; iodoform, gr. i.; and borax, gr. ii.; has been of considerable service, from its double action as sedative and antiseptic. When there is much pain on swallowing, the spraying with cocaine (4 or 10 per cent. solutions) has proved of considerable value, and it has the advantage that it can be given more frequently than sprays or powders containing morphia.

